# Periodic Hemorrhage from the Face

**Published:** 1851-06

**Authors:** 


*Periodic Hemorrhage from the Face.*—Dr. Chrestien, of Montpelier, relates the case of a young lady who had been sent to the baths at Rennes for the regulation of her menses, which had never appeared through the genital organs, but through the pores of the skin of the cheeks. Drops of blood appear upon these parts, return very soon after being wiped off, and continue in this way until the loss amounts to about 100 or 120 grammes of blood per day. This hemorrhage has already appeared several times at intervals resembling those of natural menstruation, and seems to supply its place.—*Ibid.*

